# Associations Between Maternal Post-traumatic Stress Disorder and Traumatic Events With Child Psychopathology: Results From a Prospective Longitudinal Study

**DOI:** 10.3389/fpsyt.2021.718108

**Published:** 2021-08-30

**Authors:** Jennifer Glaus, Virginie Pointet Perizzolo, Dominik A. Moser, Marylène Vital, Sandra Rusconi Serpa, Sébastien Urben, Kerstin J. Plessen, Daniel S. Schechter

**Affiliations:** ^1^Division of Child and Adolescent Psychiatry, Department of Psychiatry, Lausanne University Hospital (CHUV) and University of Lausanne, Lausanne, Switzerland; ^2^Department of Psychiatry, Faculty of Medicine, University of Geneva, Geneva, Switzerland; ^3^Department of Child and Adolescent Psychiatry, University of Geneva Hospitals, Geneva, Switzerland; ^4^Faculty of Psychology, University of Geneva, Geneva, Switzerland; ^5^Department of Child and Adolescent Psychiatry, New York University Grossman School of Medicine, New York, NY, United States

**Keywords:** interpersonal violence, post-traumatic stress disorder, child psychopathology, traumatic events, children, mothers

## Abstract

**Introduction:** Exposure to interpersonal violence (IPV) can lead to post-traumatic stress disorder (PTSD) in mothers, and in turn adversely affect the mother-child relationship during early development, as well as the mental health of their children. Our objectives are to assess: (1) the association of maternal IPV-PTSD to child psychopathology, (2) the association of maternal IPV independently of PTSD to child psychopathology, and (3) the relationship between child exposure to violence to the psychopathology of these children.

**Methods:** We used data from the longitudinal Geneva Early Childhood Stress Project. The sample included 64 children [mean age at Phase 1 = 2.4 (1.0–3.7) years] of mothers with or without IPV-PTSD. Data on mothers was collected during Phase 1, using the Clinical Administered PTSD Scale (CAPS), the Brief Physical and Sexual Abuse Questionnaire (BPSAQ) and the Conflict Tactics Scale (CTS2). Modules of a semi-structured diagnostic interview, and the Violence Exposure Scale were used to collect information on child at Phase 2, when children were older [mean age = 7.02 (4.7–10)].

**Results:** A higher CAPS score in mothers when children were toddler-age was associated with an increased risk of symptoms of attention deficit/hyperactivity disorder (ADHD; *β* = 0.33, *p* = 0.014) and PTSD in school-age children. The association between maternal IPV-PTSD and child PTSD (*β* = 0.48, *p* < 0.001) symptoms remained significant after adjustment for potential confounders. Among children, exposure to violence was associated with an increased risk of symptoms of generalized anxiety (*β* = 0.37, *p* = 0.006), major depressive (*β* = 0.24, *p* = 0.039), ADHD (*β* = 0.27, *p* = 0.040), PTSD (*β* = 0.52, *p* < 0.001), conduct (*β* = 0.58, *p* = 0.003) and oppositional defiant (*β* = 0.34, *p* = 0.032) disorders.

**Conclusion:** Our longitudinal findings suggest that maternal IPV-PTSD during the period of child development exert an influence on the development of psychopathology in school-aged children. Mothers' IPV was associated with child psychopathology, independently of PTSD. Child lifetime exposure to violence had an additional impact on the development of psychopathology. Careful evaluation of maternal life-events is essential during early childhood to reduce the risk for the development of child psychopathology. Early efforts to curb exposure to violence in children and early intervention are both needed to reduce further risk for intergenerational transmission of trauma, violence, and related psychopathology.

## Introduction

Mental disorders in children are a public health issue. They are one of the highest sources of premature mortality worldwide and the leading cause of disability in children (1). Targeted prevention and avoidance of risk factors during the period of early development could prevent the serious long-term health consequences of these disorders in children. Previous literature showed multiple complex risk factors for mental disorders in children, such as parental mental disorders (2, 3), childhood traumatic experience (4, 5), poverty (5) and genetics (e.g., familial transmission) (6, 7). Additionally, interpersonal violence (IPV) is also a risk factor that can be preventable through targeted intervention strategies (1).

In particular, exposure to interpersonal violence (IPV, e.g., childhood domestic violence exposure, sexual or physical abuse, experience of domestic violence in relationships of adult couples), are highly related to subsequent post-traumatic stress disorder (PTSD) in adults and children (8–11). Moreover, although exposure to IPV during childhood has been less explored, previous results have shown an association with other mental health disorders, such as mood and anxiety (10, 11). A recent mini review on early life exposure to IPV reported increased developmental and behavioral problems in children exposed to any type of IPV compared to unexposed children (10). Studies also showed that both being a victim and witnessing violence during childhood is a risk factor for subsequent neuropsychiatric disorders (12), with one study reporting victimization to violence to have the strongest effect compared to witnessing (13).

When mothers suffer from IPV related PTSD (IPV-PTSD), this not only has an impact on the mothers themselves and their interactive behavior and physiology (14–16), but can have consequences on the mental health of their children too (17–19). The impact of maternal disorders on the child could occur both directly and indirectly, for example through the effects of maternal psychopathology on parenting stress and, in turn, on the parent-child relationship (15, 20–23). Potentially because of this, maternal morbidity can impact the child's behavior toward others (24).

A recent review of the literature including 12 studies found an overall consistent association between maternal childhood experience of IPV during childhood and subsequent psychopathology in their children (4). Specifically, externalizing problems were more frequent in children of mothers with childhood maltreatment compared to internalizing problems (4). Additionally, children of mothers with IPV-PTSD displayed increased internalizing and externalizing symptoms, as well as PTSD (25–29). However, the precise impact of specific maternal maltreatment types and related psychopathology (i.e., PTSD, major depressive disorder, and related comorbidity) on the mental health of children during formative development has not yet been clarified in a longitudinal design. We hypothesize different associations between maternal maltreatment and child psychopathology, depending on maltreatment types. Another review of the literature including 11 studies reports an association between maternal violence exposure during pregnancy and child developmental disorders (e.g., autism spectrum disorder), as well as internalizing and externalizing behaviors (30). However, studies using different designs and methodologies were included and current evidence remains limited. Moreover, it is also important to consider maternal traumatic events experienced since the time of the child's birth, as this is a sensitive period to create a healthy mother-child relationship (31). Accordingly, the objectives of this study were to:

1. examine longitudinally the effect of maternal IPV-PTSD when children were ages 1-3.5 years old on specific child psychopathology (i.e., anxiety, behavioral problems, and major depressive symptoms) when children were 5-9 years old;
2. investigate the associations of maternal lifetime experience of IPV events on child psychopathology;
3. assess the association between children's lifetime exposure to violence and the development of child psychopathology.

## Materials and Methods

The Institutional Ethics' Committee of the Geneva University Hospital approved this study and is in accordance with the Helsinki Declaration (32). All participants' parents provided written informed consent for the study protocol. All study data were de-identified as much as possible. Study data were identified using a study subject code and the connection to identifiable data was accessible only to limited personnel under signed agreement that protected participants' anonymity.

### Study Sample

The present study analyzed data from the Geneva Early Childhood Stress Project (GECS-Pro), a longitudinal study composed of two phases examining the effects of maternal IPV-PTSD on their children. The sample was derived by posting flyers at the Geneva University Hospital, community centers, domestic violence agencies, and shelters, the latter in order to oversample for domestic violence-exposed mothers. The study fielded calls from interested mothers. In such cases, the protocol was explained in short, including demands and the benefits, the latter of which -among other things- included monetary remuneration of up to 300 CHF for the overall study (which included neuroimaging), as well as a referral. A return was given in case the clinicians responsible for the assessments and the supervisor of the study thought it necessary. In some cases, an assessment of the mother-child interaction was given using video feedback. Phase 1 was conducted when children were 12–42-months-old and the focus was on the potential effects of a maternal caregiving environment as affected by maternal IPV-PTSD on the parent-child relationship during formative periods for the development of emotion regulation. Phase 2 followed the children into school-age ages 5–9-years-old during which time sufficient self-regulation of emotion is expected for the child to be able to learn and socialize. Phase 1 inclusion criteria included dyads of biological mothers and children (between 12 and 42 months old), with mother willingness to consent to have their child participate in the study, and an ability to speak French. Exclusion criteria included mothers with active substance abuse or psychotic disorder. We did not include fathers as some mothers were living under order of protection and in anonymous shelters. The same mother-child dyads were included in Phase 2 when children were aged between 5 to 9 years old. A total of 67 mother-child dyads participated at Phase 2, out of the 84 dyads that participated at Phase 1 (80% participation). The two groups (dropouts and Phase 2 sample) did not differ in terms of parent or child age, child gender, socio-economic status, or number of traumatic life-events. Among them, three mother-child dyads were excluded due to incomplete data on child psychopathology. The final sample used to examine the associations between maternal IPV-PTSD and traumatic events with child psychopathology consisted of 64 children [mean age = 2.4 (1.0–3.7) years] of mother with or without a diagnosis of IPV-PTSD. The sample was a sample of convenience, in the sense that it depended on the original sample, which was not designed for this specific study, but rather for the needs of fMRI studies that were part of phase 1.

### Measures

#### Child Psychopathology and Exposure to Violence

Mental disorder symptoms in children at Phase 2 were assessed using a French translation version of the semi-structured interview Schedule for Affective Disorders and Schizophrenia for School-aged Children—Epidemiologic version (K-SADS-E) (33). Reliability of the French version has been tested previously (34). For the present study, only certain modules of the interview were used, due to time constraints. We were thus able to use the number of symptoms of several disorders (instead of diagnosis), including separation anxiety disorder (SAD), general anxiety disorder (GAD), major depressive disorder (MDD), attention deficit/hyperactivity disorder (ADHD), PTSD, conduct disorder (CD), and oppositional defiant disorder (ODD).

We assessed exposure to violence at Phase 2 using the French translated version of the Violence Exposure Scale (VEX) (35, 36). Children were asked how often they had experienced or witnessed violent events in three different settings (home, school and in the street), with the help of cartoon style pictures representing a child. We created an overall variable corresponding to the number of settings in which the child was exposed to violence either as victim or as witness. This instrument has an acceptable to good reliability (37).

#### Maternal Measures

Maternal sociodemographic variables (age and SES) were collected at Phase 1, using the Geneva Socio-Demographic Questionnaire (38), which was adapted from the Structured Clinical Interview for the DSM IV (39). Measures on maternal psychopathology were assessed at Phase 1, as children were considered to be in a sensitive period of development, which allowed for the prediction of psychopathological symptoms in children. We collected information on maternal IPV-PTSD using the structured interview Clinical Administered PTSD Scale (CAPS) at Phase 1. The CAPS includes 30 items, which correspond to the DSM-IV diagnosis for PTSD, and gives a total symptom severity score. It is the gold standard in PTSD assessment, with high sensitivity (90%) and specificity (95%), as well as a Cronbach's alpha coefficient of 0.97 (40). Women were categorized as PTSD if they had at significant symptoms (score >40) and fulfilled criteria for lifetime PTSD as indicated in DSM IV, which was the most current DSM version at the start of phase 1 (when the study was implemented). History of traumatic events in mothers was measured using the Brief Physical and Sexual Abuse Questionnaire (BPSAQ) (41) at Phase 1, with a focus on the severity of maternal violent trauma history (21). We created three variables from the questionnaire: domestic violence, physical and sexual abuse. Additionally, mothers completed a French version of the Revised Conflict Tactics Scale 2 Short Version (CTS2) (42) at baseline that was enlarged with 6 items from the larger CTS (43). This version of the CTS2 includes 16 items and assesses the violence in the mother's current/most recent romantic relationship, with each item further divided into acts perpetrated by the partner or by the mother herself. In line with the belonging of these items within the literature (42, 43) the items were subdivided into three subscales: physical assault in the relationship, psychological aggression in the romantic relationship (without physical violence), and reasoning and other verbal arguing behaviors in the romantic relationship.

#### Covariates

Data were collected on mother and child's age and sex, socio-economic status, as well as depressive symptomatology in mothers. We collected information on sociodemographics using the Geneva Socio-Demographic Questionnaire (GSQ) (38), the family socio-economic status (SES) using the Largo Index (44) and the Beck Depression Inventory II (BDI) (45) to map depressive symptoms.

### Statistical Analysis

Statistical analyses were conducted using the Statistical Analysis System (SAS Institute Inc., Cary, NC, USA) version 9.4 for Windows. Continuous independent variables were standardized. We generated descriptive data for demographic characteristics, maternal psychopathology, exposure to traumatic events, as well as child psychopathology and exposure to violence. Associations between maternal PTSD and traumatic events at Phase 1 with child psychopathology (number of symptoms) at Phase 2 were determined using Poisson regression models. Two models of increasing complexity were computed for each child disorder. Model 1 included either maternal PTSD or a traumatic event at a time as the independent variable at Phase 1, adjusted for sex and age of the child and SES in mothers. Model 2 was further adjusted for maternal depressive symptoms in order to determine the specific effect of maternal depression on child psychopathology.

Associations between exposure to violence with child psychopathology were determined using Poisson regression models. Two models were computed. Model 1 included child exposure to violence as independent variable, adjusted for sex and age of the child, as well as SES in mothers. Model 2 was further adjusted for maternal depressive symptoms and PTSD. We employed the Benjamini-Hochberg false discovery method (46) to correct for multiple tests.

Additionally, complementary models were performed to test interactions between maternal PTSD and child exposure to violence upon child psychopathology, whenever we prior found a link between maternal PTSD and child psychopathology.

## Results

### Sample Characteristics

The mean age for mothers was 34.59 (s.d. = 6.02) years old and 62.50% of them experienced PTSD at Phase 1 (see Table 1). In addition, 40.32% of the mothers in our sample experienced domestic violence during their childhood. Concerning children, 43.75% of them were girls. The mean age at Phase 1 was 2.40 (s.d. = 0.70) and 7.02 (s.d. = 1.13) during Phase 2. On average, children had one symptom per psychiatric disorder, with SAD, MDD and PTSD being the more frequent, and CD and ODD being the less frequent.

**Table 1:** Sample characteristics (*n* = 64).

	**Mean (SD)**	**Min-max**
**Maternal variables at Phase 1**		
Age, years	34.59 (6.02)	22–47
SES	5.07 (1.91)	2–9
CAPS total symptom severity score	59.42 (35.34)	16–129
Depressive symptoms	9.17 (7.56)	0–34
**Childhood interpersonal violence exposure severity**		
Domestic violence, % (n)	40.32 (25)	–
Physical abuse	2.38 (2.85)	0–10
Sexual abuse	1.11 (2.34)	0–7
**Violence in the romantic relationship**		
Violence by partner, % (n)	23.44 (15)	–
Violence by self, % (n)	25.00 (16)	–
Psychological aggression by partner	5.86 (5.35)	0–19
Psychological aggression by self	5.13 (4.60)	0–22
Reasoning by partner	12.48 (5.35)	0–22
Reasoning by self	14.84 (7.48)	0–59
**Child variables at Phase 2**		
**Sex, % (** ***n*** **)**		
Girls	43.75 (28)	–
Boys	56.25 (36)	–
Age, years (Phase 1)	2.40 (0.70)	1.00–3.67
Age, years (Phase 2)	7.02 (1.13)	4.67–10.00
**Number of symptoms**		
Separation anxiety disorder	1.95 (1.61)	0–5
General anxiety disorder	1.16 (1.20)	0–4
Major depressive disorder	1.52 (1.51)	0–6
Attention Deficit/Hyperactivity Disorder	1.13 (1.37)	0–4
Post-traumatic stress disorder	2.35 (3.31)	0–13
Conduct disorder	0.61 (1.05)	0–4
Oppositional defiant disorder	0.78 (1.0)	0–3
**Exposure to violence**		
Number of settings in which the child was exposed to violence either as victim or as witness	6.35 (2.33)	1–12

*SES, socio-economic status; CAPS, clinical administered PTSD scale*.

### Associations Between Maternal PTSD and History of Traumatic Events With Child Psychopathology

When models were only adjusted for socio-demographic variables, maternal PTSD at baseline was associated with an increased number of ADHD and PTSD symptoms in children at Phase 2 (Table 2). These associations remained significant after further adjustment for maternal depressive symptoms, but only the association between maternal PTSD and increased PTSD symptoms in children remained significant after correction for multiple testing. Regarding the associations between maternal traumatic events during childhood at Phase 1 and child psychopathology at Phase 2, we found that maternal childhood exposure to domestic violence predict an increase in the number of PTSD symptoms in the children. However, after adjustment for potential confounders the association did not remain statistically significant. Moreover, maternal childhood exposure to domestic violence did not predict an increase in the number of other symptoms in the children. However, maternal childhood physical abuse was associated with increased MDD symptoms and maternal history of childhood sexual abuse was associated with increased ADHD symptoms in children. Those associations did not remain significant after further adjustment for potential confounders. Concerning the associations between mothers' adult history of violence in their romantic relationships with child psychopathology at school-age, maternal-reported partners' verbal violence (i.e., non-physical violence), as well as maternal self-reported verbal violence and psychological aggression (both perpetrated by the partner or the mother) were associated with increased PTSD symptoms in children. When the models were further adjusted for potential confounders, the association between partners' physical violence and non-physical violence remained significantly associated with child PTSD symptoms. However, none of these associations remained significant after correction for multiple testing.

**Table 2:** Associations between maternal PTSD and traumatic events at Phase 1 with child psychopathology at Phase 2 (*n* = 64).

	**Number of symptoms in children at Phase 2**													
	**SAD**		**GAD**		**MDD**		**ADHD**		**PTSD**		**CD**		**ODD**	
**Maternal variables**	***β***	***P***	***β***	***P***	***β***	***P***	***β***	***P***	***β***	***P***	***β***	***P***	***β***	***P***
**Model 1**														
PTSD	0.16	*0.108*	0.04	*0.729*	−0.03	*0.814*	**0.33**	***0.014***	**0.49** ^*^	***<0.001***	−0.15	*0.382*	0.06	*0.723*
**Interpersonal violence exposure severity**														
Domestic violence	0.01	*0.946*	−0.39	*0.131*	−0.16	*0.466*	−0.02	*0.949*	**0.38**	***0.027***	−0.37	*0.280*	−0.27	*0.382*
Physical abuse	−0.01	*0.944*	–**0.32**	***0.025***	**0.21**	***0.036***	0.11	*0.378*	0.14	*0.068*	−0.01	*0.939*	−0.22	*0.195*
Sexual abuse	0.15	*0.072*	0.21	*0.052*	−0.02	*0.885*	**0.22**	***0.046***	0.09	*0.674*	−0.00	*0.999*	−0.03	*0.854*
**Violence in the relationship**														
Violence by partner	−0.14	*0.534*	0.13	*0.635*	0.02	*0.923*	−0.13	*0.677*	0.68^*^	***<0.001***	−0.09	*0.832*	−0.70	*0.096*
Violence by self	−0.10	*0.644*	0.18	*0.495*	0.17	*0.447*	0.01	*0.967*	**0.51**	***0.003***	0.07	*0.846*	−0.49	*0.191*
Psychological aggression by partner	−0.10	*0.317*	−0.13	*0.325*	0.01	*0.894*	−0.05	*0.676*	0.31^*^	***<0.001***	−0.24	*0.203*	−0.24	*0.130*
Psychological aggression by self	−0.11	*0.249*	−0.14	*0.255*	−0.08	*0.457*	−0.07	*0.558*	**0.17**	***0.013***	0.02	*0.927*	−0.10	*0.470*
Reasoning by partner	−0.02	*0.849*	0.02	*0.870*	0.21	*0.075*	−0.18	*0.157*	–**0.21**	***0.025***	0.03	*0.849*	0.04	*0.772*
Reasoning by self	−0.12	*0.307*	0.02	*0.870*	0.11	*0.272*	−0.14	*0.370*	–**0.22**	***0.049***	−0.01	*0.946*	−0.01	*0.977*
**Model 2**														
PTSD	0.03	*0.802*	0.02	*0.901*	−0.24	*0.113*	**0.35**	***0.029***	0.48^*^	***< .001***	−0.02	*0.915*	0.14	*0.470*
**Interpersonal violence exposure severity**														
Domestic violence	−0.15	*0.442*	−0.48	*0.071*	−0.23	*0.334*	−0.27	*0.306*	0.12	*0.505*	−0.34	*0.365*	−0.41	*0.208*
Physical abuse	−0.08	*0.434*	**-0.36**	***0.013***	0.19	*0.071*	0.04	*0.750*	0.02	*0.807*	0.03	*0.865*	−0.24	*0.153*
Sexual abuse	0.11	*0.212*	0.19	*0.094*	−0.02	*0.851*	0.15	*0.208*	−0.03	*0.741*	0.01	*0.971*	−0.09	*0.581*
**Violence in the relationship**														
Violence by partner	−0.40	*0.097*	0.08	*0.797*	−0.11	*0.679*	−0.38	*0.233*	**0.43**	***0.023***	0.09	*0.838*	−0.80	*0.065*
Violence by self	−0.28	*0.195*	0.14	*0.617*	0.12	*0.611*	−0.19	*0.515*	0.27	*0.127*	0.26	*0.527*	−0.55	*0.153*
Aggressive without violence by partner	−0.19	*0.063*	−0.16	*0.229*	−0.03	*0.777*	−0.14	*0.281*	**0.21**	***0.010***	−0.19	*0.325*	−0.25	*0.110*
Aggressive without violence by self	−0.20	*0.061*	−0.17	*0.197*	−0.14	*0.224*	−0.12	*0.383*	0.11	*0.121*	0.09	*0.572*	−0.08	*0.581*
Verbal violence by partner	−0.01	*0.932*	0.04	*0.793*	0.20	*0.088*	−0.14	*0.302*	–**0.20**	***0.039***	0.06	*0.722*	0.11	*0.492*
Verbal violence by self	−0.13	*0.301*	0.03	*0.814*	0.11	*0.272*	−0.12	*0.471*	−0.22	*0.056*	0.00	0.987	0.03	*0.861*

*SAD, separation anxiety disorder; GAD, generalized anxiety disorder; MDD, major depressive disorder; ADHD, attention deficit/hyperactivity disorder; PTSD, post-traumatic stress disorder; CD, conduct disorder; ODD, oppositional defiant disorder; p, P-value*.

P-values (uncorrected for multiple testing) are in italic. Statistically significant results are in bold. Results that remained significant after FDR are indicated with a ^*^.

*Continuous independent variables were standardized*.

*Model 1: adjusted for sex, age of the child and maternal socio-economic status*.

*Model 2: model 1 further adjusted for maternal depressive symptoms*.

*Models with maternal traumatic events were further adjusted for maternal PTSD in Model 2*.

### Associations Between Exposure to Violence and Psychopathological Symptoms in Children

The number of settings in which the child was exposed to violence either as victim or as witness was associated with an increased number of symptoms of GAD, MDD, ADHD, PTSD, CD, and ODD, in both models (Table 3). Moreover, these associations remained significant after correction for multiple testing. Specifically, the increased number of settings in which the child was exposed, the increased number of psychopathological symptoms the children had. Settings included home, school and street. Potential confounders, including sex and age of the child, maternal SES, PTSD, and MDD, did not better explain these associations, and the effect size was only slightly diminished. In contrast, child exposure to violence was not associated with symptoms of SAD.

**Table 3:** Associations between children's exposure to violence and child psychopathology at Phase 2 (*n* = 59).

	**Number of symptoms in children**													
	**SAD**		**GAD**		**MDD**		**ADHD**		**PTSD**		**CD**		**ODD**	
	***β***	***P***	***β***	***P***	***β***	***P***	***β***	***P***	***β***	***P***	***β***	***P***	***β***	***P***
**Model 1**														
**Exposure to violence**	0.15	*0.118*	**0.38**	***0.004***	**0.26**	***0.024***	**0.29**	***0.018***	**0.56**	***< .001***	**0.46**	***0.011***	**0.31**	***0.041***
**Model 2**														
**Exposure to violence**	0.10	*0.302*	**0.37**	***0.006***	**0.24**	***0.039***	**0.27**	***0.040***	**0.52**	***< .001***	**0.58**	***0.003***	**0.34**	***0.032***

*SAD, separation anxiety disorder; GAD, generalized anxiety disorder; MDD, major depressive disorder; ADHD, attention deficit/hyperactivity disorder; PTSD, post-traumatic stress disorder; CD, conduct disorder; ODD, oppositional defiant disorder; p, p-value*.

*P-values (uncorrected for multiple testing) are in italic. Statistically significant results are in bold. All the results that remained significant after FDR correction*.

*Model 1: adjusted for sex, age of the child and maternal socio-economic status*.

*Model 2: model 1 further adjusted for maternal PTSD and depressive symptoms*.

### *Post-hoc* Analyses

To better understand the relationship of two of our findings, namely that (a) PTSD in mothers was associated with increased PTSD symptoms in children independently, and (b) child exposure to violence was associated with child PTSD symptoms, we performed additional analyses. Specifically, we tested: (I) whether maternal PTSD was associated with an increase in child exposure to violence, and (II) using Poisson regression, we tested whether there was an interaction between maternal PTSD and child exposure to violence upon child PTSD symptoms. When we investigated the association between maternal PTSD with child exposure to violence, the association was not statistically significant (*β* = 0.03, *p* = 0.618). However, there was an interaction between maternal PTSD and child exposure to violence on child PTSD (*β* = 0.10, *p* = 0.038). Specifically, results showed that the higher the severity of PTSD in mothers, the higher the effect of child exposure to violence on child PTSD symptoms. In the interaction model, the main effect for child exposure to violence remained significant (*β* = 0.25, *p* < 0.001), whereas the one for PTSD in mothers did not reach the statistical difference (*β* = −0.04, *p* = 0.895).

## Discussion

The present study assessed the longitudinal associations between maternal IPV-PTSD and history of traumatic events when the children were toddlers with subsequent development of psychopathological symptoms in school-aged children. Our results showed that higher maternal PTSD severity predicted an increase in psychopathological symptoms in school-aged children. In particular, higher maternal PTSD severity was associated with increased PTSD symptoms in children after adjustment for potential confounders. Additionally, an increased number of settings in which the child was exposed to violence either as victim or as witness were associated with increased psychiatric symptoms in school-aged children.

Our results are in line with previous studies on the association between maternal PTSD and child psychopathology (25, 27, 29). Our findings further showed that among all psychopathological symptoms investigated in children, PTSD symptoms are the most consistently predicted by maternal PTSD. When we further investigated which lifetime traumatic event was associated with PTSD in children, results showed that children who were exposed to violence were at increased risk for PTSD symptoms, corroborating previous findings (10). Increasing numbers of studies have examined the genetic and epigenetic risk factors involved in the pathogenesis of PTSD given that only a small percentage of those who experience traumatic events develop the disorder (47). Yet a large GWAS study put into perspective that, in reality, genetic factors likely only account for 10 to 20% of the probability that an individual will develop PTSD following a traumatic life-event (48). That being said, at least one other study has pointed to the possibility that exposure to interpersonal violence may also be linked to familial gene risk conferring up to 20% risk for experiences of physical assault (49). Indeed, children who were directly victims of violence and those who witnessed violent behaviors in their lifetime were both at increased risk of PTSD symptoms at school-age compared to children who were not exposed to violence. And so, clearly, the environmental contribution to the pathogenesis of PTSD, even if not as great as thought 25 years ago, is still relatively high compared to other psychiatric disorders. This finding is in line with a recent mini-review that concluded that exposure to any violence during early childhood can have adverse effects on the child mental health (10). Thus, as several important studies have shown that genetic and epigenetic factors are involved in intergenerational transmission of PTSD, clinical efforts should particularly focus on the prevention of PTSD symptoms and violence in children whose mother suffered from PTSD when their children were toddlers, particularly when those toddlers have been or go on to be exposed themselves to IPV. We additionally showed that exposure to violence in children was specifically associated with different psychiatric symptoms, including GAD, MDD, ADHD, PTSD, CD and ODD. This may indicate that child exposure to violence is not specifically a risk factor for increased PTSD symptoms in school-aged children, but that being victim or witness of a violence is also a risk factor for the development of other psychiatric symptoms. This result is in line with previous studies that showed increased risk of internalizing and externalizing disorders, as well as interference with development in children exposed to IPV (10).

**Figure 1:**
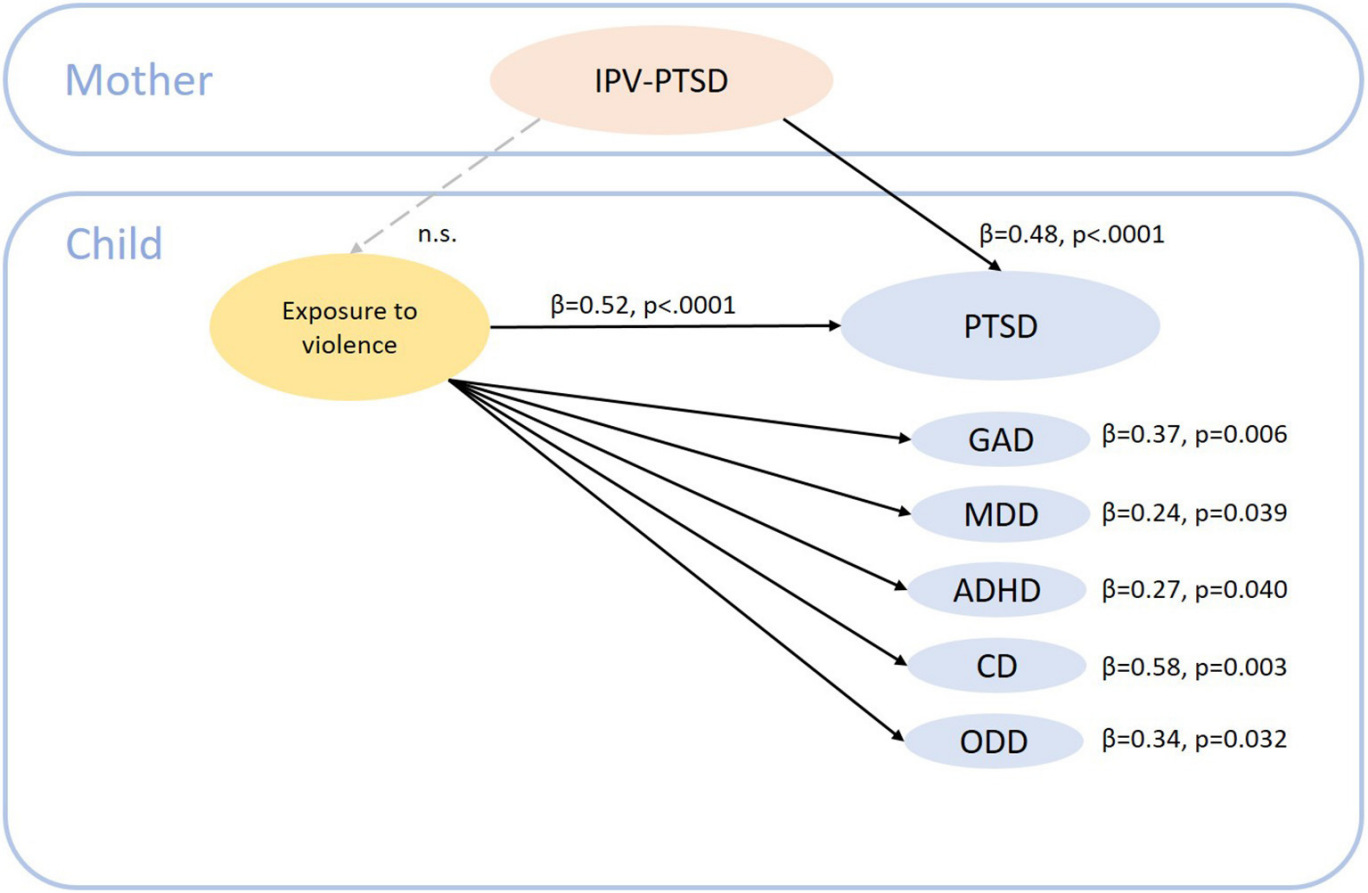
Summary of main results. IPV-PTSD, interpersonal violence related post-traumatic stress disorder; PTSD, post-traumatic stress disorder; GAD, generalized anxiety disorder; MDD, major depressive disorder; ADHD, attention/deficit hyperactivity disorder; CD, conduct disorder; ODD, opposition defiant disorder.

To our knowledge, this is the first study that investigated specific lifetime maternal traumatic events and related PTSD in association with subsequent development of psychopathological symptoms in children using a follow-up design. However, our findings also indicated that a maternal history of childhood domestic violence, physical and/or sexual abuse did not predict subsequent development of child psychopathological symptoms, independently of whether the mother also suffered from PTSD when the children were toddlers once results are corrected for multiple comparisons. Our results differ in this regard somewhat from previous studies on maternal IVP and child psychopathology (4, 30). This discrepancy could be due to methodological differences, such as the instruments used to assess psychopathological symptoms, as well as the heterogeneity of children's age recruited in these studies. Indeed, only two studies included in the systematic review of Plant et al. (4) investigated self-reported child psychopathology, whereas in the other studies, mothers reported the symptoms/disorders of their children. Additionally, the studies included in the aforementioned review and Toso's et al. (30) review, examined maternal IPV that mothers experienced in their own childhood and only maltreatment events, or during pregnancy exclusively; whereas, we considered lifetime traumatic events too. Replication of those findings in longitudinal studies which use a diagnostic instrument and a full range of maternal traumatic events across the mother's lifespan in relation to child psychopathology are needed. In *post-hoc* analyses, we aimed to elucidate the underlying mechanism for the association between maternal IPV-PTSD when children were toddlers and increased PTSD symptoms in school-aged child (c.f. Figure 1). Since child lifetime exposure to violence was also associated with increased PTSD symptoms, we looked at the interaction between IPV-PTSD and exposure to stress. The result demonstrated that, in our sample, the associations between maternal IPV-PTSD and child PTSD could be moderated by the child's exposure to violence. However, further studies are needed to better elucidate the complex mechanism of how IPV-PTSD in mothers influences the development of the child's mental health.

Strengths of this study include its characterization of psychopathological symptoms, including anxiety, depressive and behavioral symptoms, as well as the direct clinical interview for the assessment of psychopathological symptoms, and the longitudinal study design. Finally, to our knowledge, this is the first study to investigate the association of specific maternal traumatic events in association with a range of several psychopathological symptoms.

Some limitations for the present study should also be noted. First, although we used a clinical interview, we used only the screening questionnaire of the K-SADS and only for selected modules. We therefore reported only the number of symptoms, as reported by the child on the questionnaire. Clinical diagnosis by the K-SADS was not possible to assess due to incomplete information. Clinicians, however, did make a provisional clinical diagnosis, a best estimate based on the screening questionnaire and their own assessments of children and their mothers. However, in the interest of rigor, we did not report those diagnoses in this paper. For this reason, our results should be interpreted in the context of this limitation. Second, children reported on their psychopathological symptoms retrospectively, and mothers reported on their traumatic events retrospectively, which may have diminished the accuracy of the data due to recall biases. Future studies may prospectively follow mothers newly exposed to traumatic events. Third, we cannot generalize those findings to maternal PTSD related to non-violent and or single-incident trauma. Evaluation of the extent to which other traumatic events, such as terrorist events, or wars, differ among mothers and the psychopathology of their children will facilitate our ability to elucidate the mechanism underlying maternal PTSD and child psychopathology. Fourth, we did not include information from the fathers or other figures of attachment. Future studies should take into account this information in order to better understand if the presence/absence of the father could have an influence on child psychopathology.

## Conclusions

Our longitudinal findings suggest that maternal IPV-PTSD during the period of child development (i.e., toddlerhood) exert an influence on the development of psychopathology in school-aged children. It is also conceivable that maternal IPV-PTSD, particularly when comorbid with major depressive symptoms, may contribute to the progression of early childhood expression of symptoms that we have described in a previous paper as a dysregulated “attachment disturbance” or “secure base distortion” (50, 51) to separation anxiety disorder at school-age. Moreover, mothers' IPV (i.e., partner violence) was associated with child psychopathology, independently of PTSD. Child lifetime exposure to violence has an impact on the development of psychopathology too. Careful consideration of maternal life-events and treatment of IVP-related psychopathology (i.e., PTSD and depression) are likely to interrupt the development of child psychopathology. Additionally, preventive efforts on the exposure to violence in children are needed to reduce the effects of violence on the child development.

## Data Availability Statement

The raw data supporting the conclusions of this article will be made available by the authors, without undue reservation.

## Ethics Statement

The studies involving human participants were reviewed and approved by The Institutional Ethics' Committee of the Geneva University Hospital. Written informed consent to participate in this study was provided by the participants' legal guardian/next of kin.

## Author Contributions

JG undertook the main literature search, contributed to the interpretation of the data, and drafted the manuscript. DM contributed to the interpretation of the data and critically revised the drafts for important intellectual content. VP, MV, SR, SU, and KP critically revised the drafts for important intellectual content. DS designed the study, supervised data collection, data management and the statistical analysis, and contributed to the interpretation of the data and critically revised the drafts. All authors contributed to the article and approved the submitted version.

## Conflict of Interest

The authors declare that the research was conducted in the absence of any commercial or financial relationships that could be construed as a potential conflict of interest.

## Publisher's Note

All claims expressed in this article are solely those of the authors and do not necessarily represent those of their affiliated organizations, or those of the publisher, the editors and the reviewers. Any product that may be evaluated in this article, or claim that may be made by its manufacturer, is not guaranteed or endorsed by the publisher.
